# Survival of the Fittest: Positive Selection of CD4+ T Cells Expressing a Membrane-Bound Fusion Inhibitor Following HIV-1 Infection

**DOI:** 10.1371/journal.pone.0012357

**Published:** 2010-08-23

**Authors:** Janine Kimpel, Stephen E. Braun, Gang Qiu, Fay Eng Wong, Michelle Conolle, Jörn E. Schmitz, Christian Brendel, Laurent M. Humeau, Boro Dropulic, John J. Rossi, Annemarie Berger, Dorothee von Laer, R. Paul Johnson

**Affiliations:** 1 Angewandte Virologie und Gentherapie, Chemotherapeutisches Forschungsinstitut Georg-Speyer-Haus, Frankfurt, Germany; 2 Division of Immunology, New England Primate Research Center, Harvard Medical School, Southborough, Massachusetts, United States of America; 3 Division of Viral Pathogenesis, Department of Medicine, Beth Israel Deaconess Medical Center, Boston, Massachusetts, United States of America; 4 VIRxSYS Corporation, Gaithersburg, Maryland, United States of America; 5 Division of Molecular Biology, Beckman Research Institute of the City of Hope, Duarte, California, United States of America; 6 J.W. Goethe University Hospital, Institute for Medical Virology, Frankfurt, Germany; 7 Sektion Virologie, Medizinische Universität Innsbruck, Innsbruck, Austria; 8 Ragon Institute of MGH, Massachusetts Institute of Technology, and Harvard, and Infectious Disease Unit, Massachusetts General Hospital, Charlestown, Massachusetts, United States of America; Institut Pasteur, France

## Abstract

Although a variety of genetic strategies have been developed to inhibit HIV replication, few direct comparisons of the efficacy of these inhibitors have been carried out. Moreover, most studies have not examined whether genetic inhibitors are able to induce a survival advantage that results in an expansion of genetically-modified cells following HIV infection. We evaluated the efficacy of three leading genetic strategies to inhibit HIV replication: 1) an HIV-1 *tat/rev*-specific small hairpin (sh) RNA; 2) an RNA antisense gene specific for the HIV-1 envelope; and 3) a viral entry inhibitor, maC46. In stably transduced cell lines selected such that >95% of cells expressed the genetic inhibitor, the RNA antisense envelope and viral entry inhibitor maC46 provided the strongest inhibition of HIV-1 replication. However, when mixed populations of transduced and untransduced cells were challenged with HIV-1, the maC46 fusion inhibitor resulted in highly efficient positive selection of transduced cells, an effect that was evident even in mixed populations containing as few as 1% maC46-expressing cells. The selective advantage of the maC46 fusion inhibitor was also observed in HIV-1-infected cultures of primary T lymphocytes as well as in HIV-1-infected humanized mice. These results demonstrate robust inhibition of HIV replication with the fusion inhibitor maC46 and the antisense Env inhibitor, and importantly, a survival advantage of cells expressing the maC46 fusion inhibitor both *in vitro* and *in vivo*. Evaluation of the ability of genetic inhibitors of HIV-1 replication to confer a survival advantage on genetically-modified cells provides unique information not provided by standard techniques that may be important in the *in vivo* efficacy of these genes.

## Introduction

Even though highly active antiretroviral therapy (HAART) has been remarkably successful in lowering the viral load in patients with HIV-infection [1], an increasing prevalence of resistant viruses [2], HAART failure [3], and a significant incidence of serious side effects [4] have provided the impetus to develop complementary therapies using genetic inhibitors of HIV-1 replication. With increased understanding of the molecular basis for HIV replication, a diverse range of genetic strategies able to inhibit HIV-1 replication *in vitro* has emerged. These genetic strategies include RNA inhibitors (i.e., ribozymes, decoys, small inhibitory RNAs, and antisense molecules), protein-based inhibitors (i.e., intracellular antibodies or dominant negative inhibitors), as well as zinc-finger nucleases that knockout host genes critical for HIV replication [5]–[8]. Although many genetic inhibitors have been demonstrated to mediate potent inhibition of HIV-1 replication [9]–[12], inhibition of viral replication has generally been evaluated using *in vitro* conditions in which >95% of cells express the inhibitor under study, a highly artificial setting given the challenges of attaining levels of even 5% to 10% genetically-modified CD4^+^ T cells *in vivo*.

A number of different criteria have been proposed for the selection of genetic inhibitors for advancement to human clinical trials, including potency of inhibition, lack of immunogenicity, lack of toxicity, and the propensity for evolution of resistant viruses [5], [6]. An additional, and perhaps underappreciated, criterion is the stage of the viral life cycle at which inhibition occurs. Inhibition of viral gene expression by inhibitors that block later stages of the viral life cycle (e.g. by blocking the function of viral proteins such as Tat or Rev) will not prevent transduced cells from becoming infected, while inhibitors that block early events in the viral life cycle prior to integration can prevent infection. Mathematical modeling has predicted that post-integration inhibitors support the accumulation of cells carrying an integrated provirus, ultimately resulting in an accumulation of HIV-1-infected cells that counteracts the antiviral effect [13]. In contrast, early inhibitors, even those with lower potency, are predicted to exert a systemic antiviral effect and to be able to mediate expansion of transduced cells able to resist HIV infection.

Translation of genetic inhibitors of HIV-1 replication into successful therapies has been limited by the disappointing rates of gene transfer to hematopoietic cells and by the relatively low rates of genetically-modified T cells that have been achieved *in vivo*. For adoptive T cell transfer studies of genetic inhibitors of HIV-1 infection, although *in vitro* transduction efficiencies resulting in more than 1 vector copy per cell have been obtained [14], after infusion into patients, the frequency of vector-containing CD4^+^ T cells *in vivo* has generally been in the range of 0.01% to 1% [14]–[18]. For trials of hematopoietic stem cell gene therapy for AIDS, levels of gene marking in CD4^+^ T cells *in vivo* after transduction with gammaretroviral vectors have been disappointingly low, typically 0.01% or less [19], [20]. At these low levels of gene marking, inhibition of HIV-1 replication in the small fraction of cells containing an inhibitory gene is unlikely to have a significant impact on either viral replication or immune reconstitution. However, if cells that contain a genetic inhibitor are able to proliferate and survive preferentially compared with unmodified cells, a vastly different scenario emerges—a progressive repopulation of the immune system with cells genetically resistant to HIV infection. A compelling proof-of-principle demonstration of this approach lies in the report of a successful transplant of an HIV-1-infected individual with bone marrow from a donor with a mutation in the HIV-1 coreceptor CCR5, which resulted in a repopulation of peripheral CD4^+^ T cells with donor cells resistant to HIV-1 infection, thereby allowing the discontinuation of antiretroviral therapy without viral rebound [21]. However, given the relatively low prevalence of bone marrow donors who are homozygous for the Δ32 CCR5 deletion (∼1% in Caucasian populations) [22] as well as the risks associated with allogeneic bone marrow transplantation, there is a compelling need for alternative strategies to induce resistance of hematopoietic cells to HIV-1 infection.

Here, we compared three HIV-specific inhibitor genes for their potency of viral inhibition and for their ability to confer a selective advantage following HIV-1 infection *in vitro* and *in vivo*. The membrane-anchored C46 peptide (maC46) [9], [23], which blocks viral fusion and entry, provided the strongest inhibition of viral replication and, importantly, a strong selective advantage for transduced cells, both *in vitro* and in immunodeficient mice transplanted with human T cells. In contrast, a long RNA antisense sequence targeting the HIV-1 envelope gene provided very strong inhibition of viral replication, but transduced cells did not exhibit a strong survival advantage *in vitro*. A short hairpin (sh) RNA inhibitor targeting the HIV-1 *tat* and *rev* genes provided modest inhibition of viral replication, coupled with an inconsistent selective advantage. Inhibitors of HIV-1 replication able to confer a survival advantage may have distinct advantages for clinical use, and these data advocate for the continued development of the maC46 peptide inhibitor as a genetic therapy strategy for AIDS.

## Results

### Genetic inhibitors of HIV-1 replication

**Figure 1:**
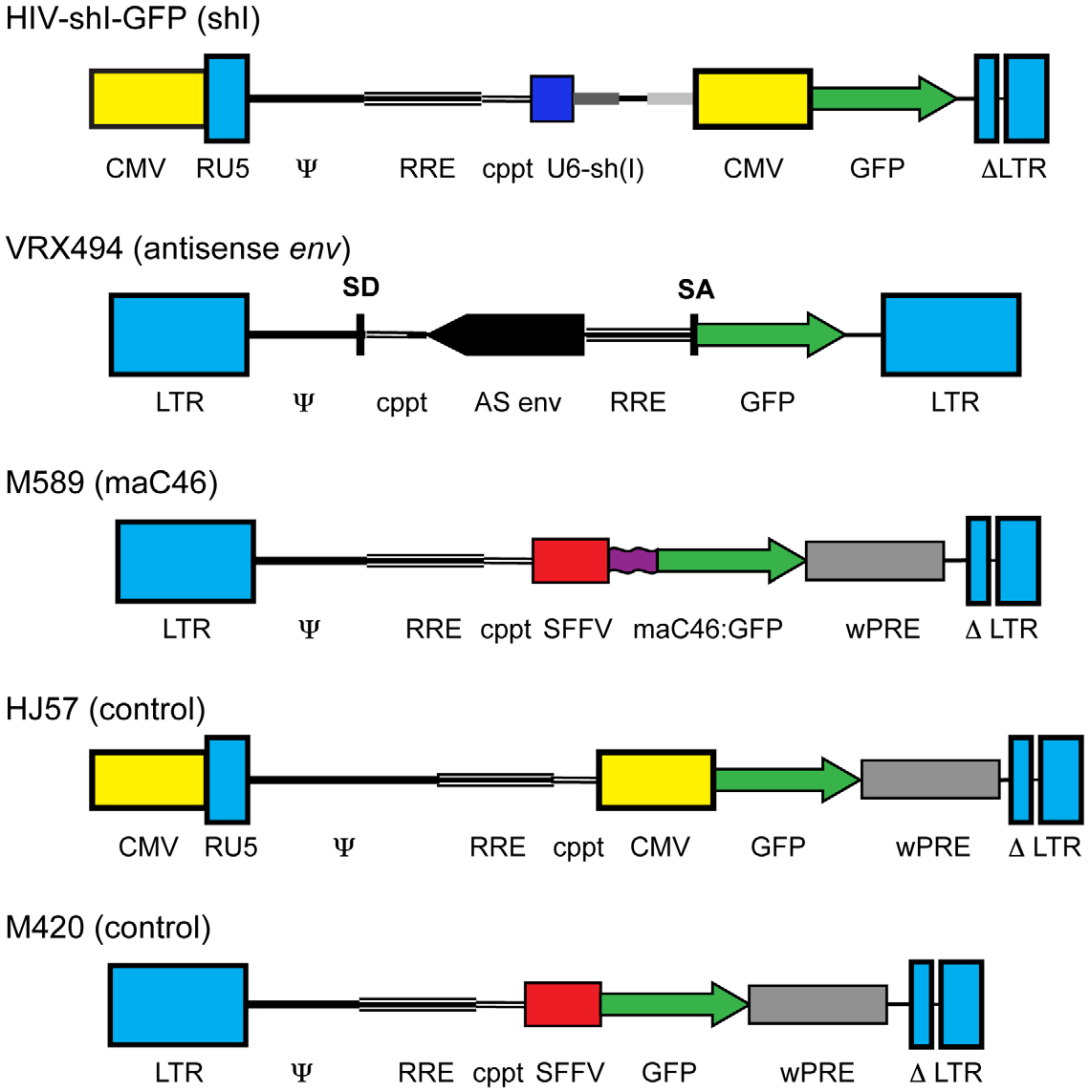
Schematic diagrams of the viral transfer plasmids. The lentiviral vectors HIV-shI-GFP, VRX494, M589, HJ57, and M420, are based on HIV-1_NL4-3_ or HIV-1_HXB2_. The viral inhibitors encoded by each of the experimental vectors are noted in parentheses. Where indicated, vectors use a heterologous CMV promoter to initiate transcription of the genomic RNA with a self-inactivating 3′ LTR. The VRX494 vector uses a functional HIV-1 LTR, which is upregulated after viral infection [36]. All vectors contain cis-acting regulatory domains (the ψ packaging signal, the central polypurine tract [cppt], the Rev response element [RRE], and, in some cases, the Woodchuck post-transcriptional regulatory element [wPRE]), and eGFP. The vector HIV-shI-GFP contains the U6 promoter regulating a shRNA targeting exon 1 of HIV-1 *tat* and *rev* (shI) [10]. The lentiviral vector VRX494 contains 937 bp of antisense (AS) HIV-1 envelope, and eGFP transcriptionally regulated by the HIV-1 LTR [32]. The vector M589 contains an internal SFFV promoter regulating expression of the C46 heptad repeat-anchored with a linker and transmembrane domain:GFP fusion protein (maC46:GFP). The control vectors HJ57 and M420 do not contain an inhibitor cassette.

We directly compared the potency of viral inhibition and the selective advantage of several lentiviral vectors expressing genetic inhibitors of HIV-1 replication: 1. HIV-shI-GFP, which contains the U6 promoter expressing a shRNA targeting exon 1 of HIV-1 *tat* and *rev*, as well as GFP expressed by an internal CMV promoter [11]; 2. M589, which contains an internal SFFV promoter expressing a membrane-anchored fusion protein (maC46) consisting of the N-terminal C46 heptad repeat from HIV-1 gp41 anchored with a linker and a transmembrane domain fused to GFP at the intracellular C-terminus [9], [23]; and 3) VRX494, which contains 937 bp of an RNA antisense HIV-1 envelope sequence and GFP transcriptionally regulated by the HIV-1 LTR [12] (Figure 1). The lentiviral vectors HJ57 and M420, which do not contain inhibitor genes but do express GFP, were included as controls. These genetic inhibitors were selected based on previous reports demonstrating their efficacy in inhibiting HIV-1 replication [9], [11], [12], their ability to target distinct sites in the retroviral life cycle, including viral entry (maC46) and as well as post-integration events (shI, VRX494), and their use, either alone [14], [18] or in combination with other genetic inhibitors [24] in human clinical trials.

### The fusion inhibitor maC46 and antisense *env* VRX494 mediate potent inhibition of HIV-1 replication in transduced cells

**Figure 2:**
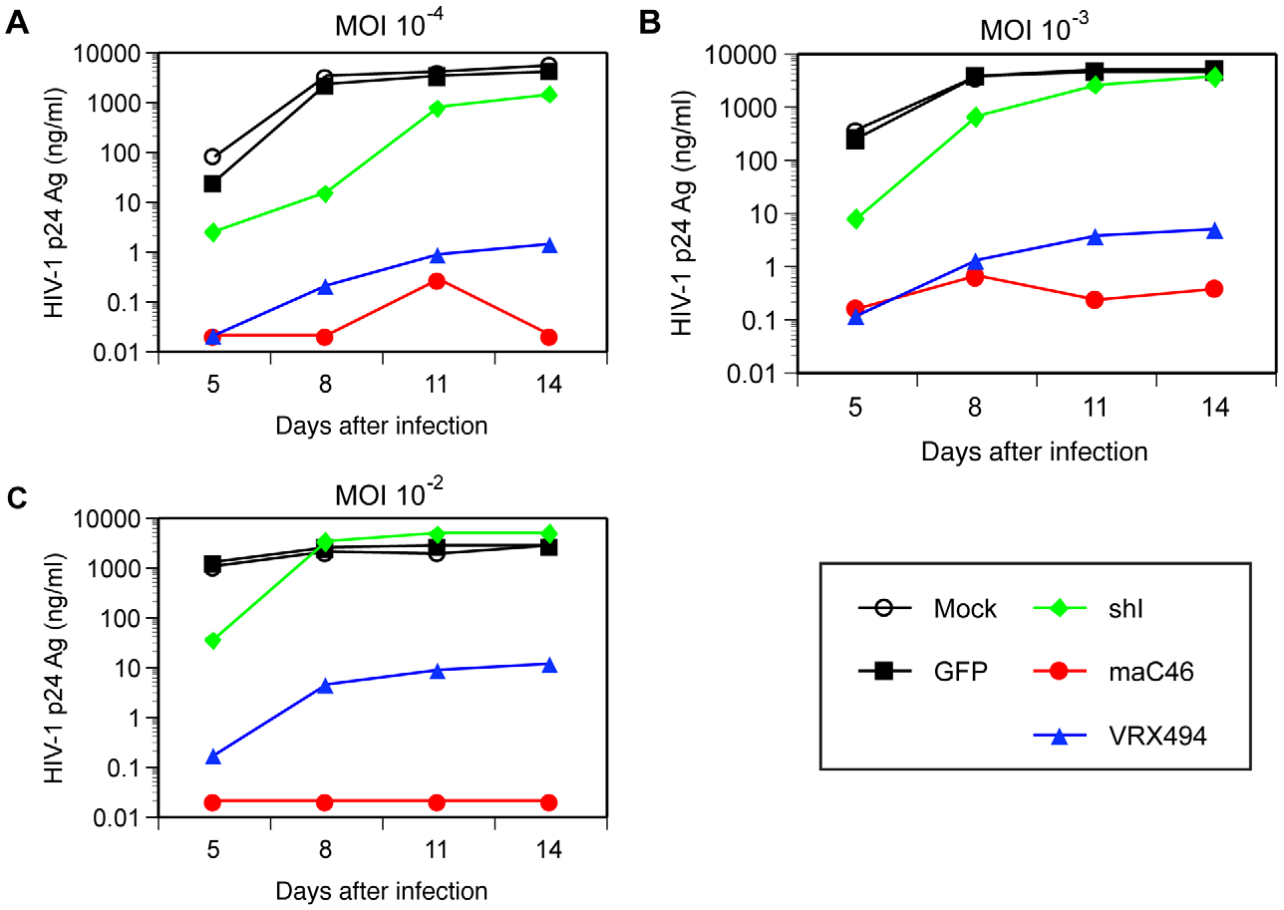
Potent inhibition of HIV-1 replication by the maC46 fusion inhibitor and antisense-Env VRX494 vectors. Non-transduced CEMx174 cells (NT) and CEMx174 cells transduced with vectors expressing the indicated inhibitor gene or a control vector (HJ57, GFP) were infected with HIV-1_NL4-3_ at MOIs of (A) 10^−4^, (B) 10^−3^, and (C) 10^−2^ TCID_50_ per cell. Viral replication was assessed by measuring HIV-1 Gag p24 antigen in cell-free culture supernatants. The minimum level of detection for HIV-1 Gag p24 was 15 pg/ml. The data are representative of at least two experiments.

To evaluate inhibition of HIV-1 replication by these three genetic inhibitors, we transduced a CD4^+^ cell line (CEMx174) with the HIV-shI-GFP, VRX494, or M589 vectors. Transduced cells were then sorted for expression of GFP, resulting in >95% GFP^+^ cells (data not shown). Sorted transduced T cells were then challenged with HIV-1_NL4-3_ at multiple MOIs. As shown in Figure 2, control T cells, either untransduced or transduced with a lentivirus expressing GFP alone, supported vigorous HIV-1 replication, with peak HIV-1 p24 antigen production exceeding 1000 ng/ml. In contrast, cells transduced with the VRX494 antisense vector or the M589 vector expressing the maC46 fusion inhibitor demonstrated quite potent inhibition of viral replication, ranging between 3 and 4 logs of inhibition compared with control cells, even at the highest MOI studied. A similar degree of inhibition mediated by these vectors was observed at MOIs of 10^−4^ and 10^−3^ TCID_50_ per cell, but at 10^−2^ TCID_50_ per cell, the highest MOI studied, maC46-expressing cells demonstrated more potent inhibition of HIV-1 replication than cells expressing the VRX494 antisense sequence. These results demonstrate that the membrane-anchored C46 peptide, which blocks fusion of viral particles to the cell membrane, and the antisense expressing vector VRX494 provide stronger inhibition of HIV-1_NL4-3_ replication than the shRNA inhibitor, a difference that was particularly evident following challenge with relatively high MOIs.

### Selective expansion of maC46-transduced cells following HIV-1 infection

**Figure 3:**
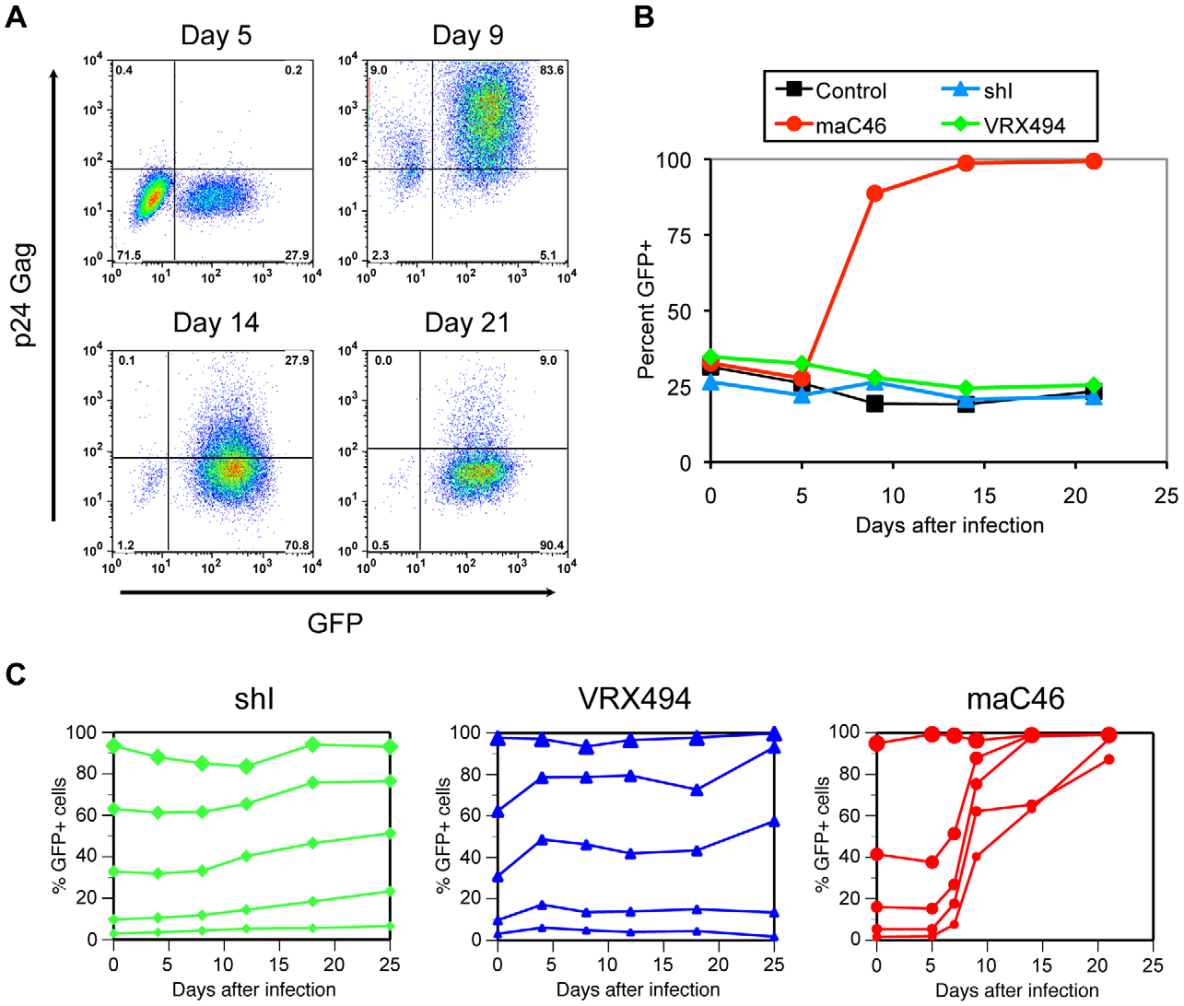
Preferential survival of cells expressing the maC46 fusion inhibitor following HIV-1 infection. A. Flow cytometric analysis for expression of GFP and HIV-1 p24 Gag at the indicated times post-infection. Non-transduced CEMx174 cells were mixed with transduced cells expressing the maC46 inhibitors such that approximately 25–30% of the cells were GFP^+^. Mixtures of cells were then infected with HIV-1_NL4-3_ at a MOI of 10^−3^ TCID_50_ per cell. Expression of HIV-1 p24 Gag and GFP were determined by flow cytometry on days 5, 9, 14 and 21. B. Survival advantage of cells expressing maC46 following HIV-1 infection. Mixtures of non-transduced CEMx174 cells and transduced cells expressing the shI, maC46, and antisense *env* inhibitors, or transduced with the HJ57 control vector (GFP) were generated such that approximately 25–30% of the cells were GFP^+^. Cells were then infected with HIV-1_NL4-3_ at MOI of 10^−3^ TCID_50_ per cell or remained uninfected. The percentage of GFP^+^ cells in the absence of HIV-1 infection was stable for each vector examined over the 21 day culture period (data not shown). C. Mixtures of non-transduced CEMx174 cells and cells transduced with vectors expressing either the sh(*tat/rev*), antisense env (VRX494), or the maC46 fusion inhibitors, were generated at the ratios indicated at time 0 and then infected with HIV-1_NL4-3_ at an MOI of 10^−3^ TCID_50_/cell. Initial mixtures of vector-transduced CEMx174 cells ranged from 1% up to 95% GFP^+^ cells. The percentage of GFP^+^ cells was determined by flow cytometry at the indicated times post-infection.

To assess the potential for the different vectors to protect transduced cells from viral cytotoxicity and therefore to provide for a selective survival advantage, non-transduced and vector-transduced CD4^+^ T cells were mixed such that approximately 25% of the cells were GFP^+^. These mixtures remained uninfected or were infected with HIV-1_NL4-3_ at an MOI of 10^−3^ TCID_50_ per cell and then followed for expression of HIV-1 p24 Gag and GFP by flow cytometry. Representative flow cytometry data are shown in Figure 3A for a mixture of transduced and untransduced cells. On day 5 after infection, the percentage of maC46-GFP-expressing cells was essentially unchanged from day 0, and only a small fraction of HIV-1 p24 cells were observed (Figures 3A and 3B). Four days later on day 9, more than 90% of the cells stained positive for HIV-1 p24 Gag and the percentage of maC46-GFP^+^ cells increased from under 30% to over 85% (Figure 3A and B). The forward/side scatter plots on day 9 showed many cells with increased granularity and decreased size characteristic of dead or dying cells, consistent with the strong cytopathicity of HIV-1 replication *in vitro* (data not shown). After 14 days, the non-transduced T cells were almost completely depleted, with only 1% of the GFP^—^ cells remaining (Figure 3A and B). Coincidently, the staining of HIV-1 p24 Gag in the GFP^+^ population also decreased to 28% (Figure 3A). By day 21 of the infection, only a few GFP^—^ cells could be detected (0.5%) and the HIV-1 p24 Gag staining in the GFP^+^ population decreased to 9%. Although a dramatic increase in the frequency of maC46-GFP-expressing cells was observed, no significant expansion of cells expressing the VRX494 antisense envelope or the shRNA inhibitor was observed (Figure 3B).

Given the low rate of transduction generally achievable in large animal models and in human clinical trials, we wished to explore the ability of these vectors to mediate a selective advantage when present at clinically relevant levels of gene marking. Therefore, mixtures of untransduced and vector-transduced T cells were generated, ranging from 1% to 60% GFP^+^ cells. Unmixed vector-transduced populations (>95% GFP^+^) were also included. These mixtures were infected with HIV-1 at an MOI of 10^−3^ TCID_50_ per cell or remained uninfected and were then followed for expression of GFP by flow cytometry (Figure 3C). As demonstrated previously in Figure 3B, cells expressing the shRNA inhibitor were not strongly selected for during HIV-1 replication, as the percent of GFP^+^ cells remained constant or only modestly increased compared to the day 0 frequencies (Figure 3C). Consistent with a previous study [25], cells expressing the antisense envelope inhibitor also demonstrated a slight increase in the percentage of transduced cells during the 25 day observation period, an effect that was most evident for the cultures containing 25% and 60% transduced cells, while no clear selective advantage was evident for the cultures containing 1% or 10% transduced cells. In sharp contrast, the percentage of maC46-expressing cells increased substantially in all mixed populations, even from a culture containing only 1% transduced cells at day 0 (Figure 3C). In the absence of HIV-1 infection, the percentage of transduced cells expressing a given transgene was relatively stable (data not shown). Thus, expression of the membrane-anchored fusion inhibitor resulted in a strong survival advantage following HIV-1 challenge *in vitro*.

### maC46-expressing cells trap HIV-1 virions at the cell surface without efficient transfer to neighboring cells

**Figure 4:**
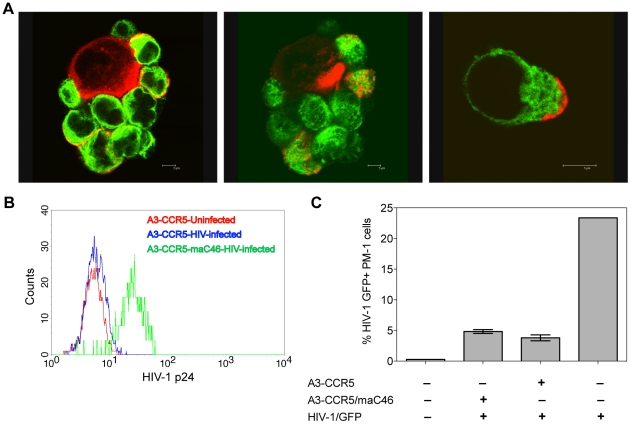
Adsorption of HIV-1 virions to the surface of transduced cells expressing maC46. A. Localization of virus in PM-1 cells transduced with M589, which expresses maC46 and GFP, was analyzed by confocal microscopy. Mixtures of maC46-GFP transduced and non-transduced cells (approximately 10% GFP^+^) were infected with HIV-1_D117/II_. After 10 days, cells were stained for HIV-1 p24 (red) and analyzed for expression of GFP (green). maC46-GFP^+^ cells show p24 signal only on the cell membrane, whereas non-transduced cells have a cytoplasmic p24 staining. B. A3-CCR5 or A3-CCR5-maC46 cells were incubated with HIV-pseudotyped viral particles. After 5 hours cells were fixed, stained for p24, and analyzed for the presence of bound viral particles. C. A3-CCR5 cells expressing maC46 were analyzed for their potential to transfer replication-incompetent lentiviral particles pseudotyped with an HIV envelope to PM-1 cells. A3-CCR5 or A3-CCR5-maC46 cells were incubated with the viral particles for 5 hours and after washing, transferred to PM-1 cells expressing RFP at a final ratio of 1∶1. GFP expression of PM-1 cells was analyzed by flow cytometry after 3 days.

Interestingly, even though the cells expressing the membrane-anchored C46 were able to mediate potent inhibition of viral replication, they also stained positive for HIV-1 p24 Gag, at least during the phase where the peak of viral replication occurred in the non-transduced cells (Figure 3A). To study if this double positive population was productively infected, we performed confocal microscopy of a mixed culture of cells expressing maC46 with untransduced cells after HIV-1 infection. As seen in Figure 4A, GFP^+^ cells expressing maC46 sequestered HIV-1 p24 antigen (red) on the cell surface but did not contain any intracellular viral antigen. In contrast, GFP^—^ p24^+^ cells contained large amounts of intracellular p24, indicating virus replication. We then asked whether virus sequestered on the surface of maC46-expressing cells was still infectious and could be transmitted to non-protected, maC46-negative cells. Such a cell-to-cell transmission could potentially enhance infection of untransduced cells, thereby accelerating virus spread in the non-protected population and the relative accumulation of the gene-protected cells in the mixed culture. Two experiments were performed to test for transmission of sequestered virus. Initially, A3-CCR5 cells expressing maC46 were incubated for 5 h at 37°C with high concentrations of a replication-deficient HIV-1 vector expressing GFP and packaged with an HIV-1 envelope (strain JR-FL). As expected, cells expressing maC46 trapped the virions on the cell surface and became clearly p24-positive as detected by FACS, while only little p24 was detected on non-transduced cells (Figure 4B). The cells were then washed and mixed with RFP-expressing PM-1 indicator cells and analyzed for virus transfer from the A3-CCR5 population to the RFP-positive indicator cell line PM-1. As a control, HIV-1 vector preparations were directly transferred to PM-1. The HIV-1/GFP vector was transferred from both the maC46-expressing and the non-modified A3-CCR5 cells, resulting in around 5% transduction of the PM-1 population (Figure 4C). However, taking into account the fact that the maC46-expressing cells trap far more HIV-1 on the cell surface than unmodified cells (as seen in Figure 4B), this observation suggests that the transfer of virus adsorbed to maC46-expressing cells is highly inefficient. Similar results were observed if the HIV-1 vector was incubated for only one hour with the A3-CCR5 cells before washing and mixing with PM-1 (data not shown).

In addition, we followed HIV-1 replication by monitoring p24 antigen in the supernatants of untransduced cells, either alone or mixed with 10%, 20% and 40% cells expressing maC46. Early virus replication kinetics were not accelerated by the addition of maC46-expressing cells (data not shown). In conclusion, maC46-expressing T cells are able to trap HIV-1 virions at the cell surface but do not efficiently transfer virus to non-transduced neighboring T cells.

### Survival of maC46-expressing primary human lymphocytes following HIV-1 infection

**Figure 5:**
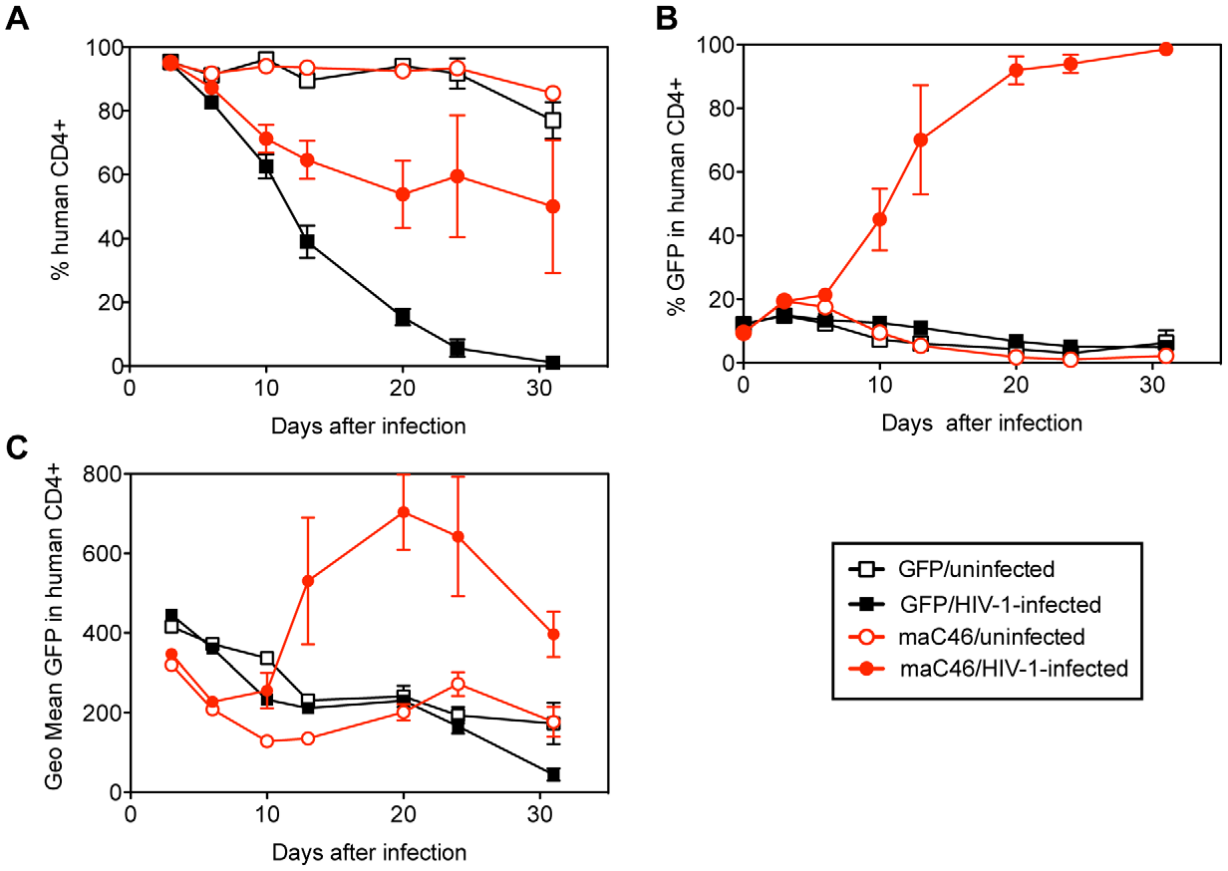
*In vitro* selection of maC46-expressing primary human T cells. Human CD4^+^ T cells were transduced with either a control lentiviral vector expressing GFP alone (M420) or a vector expressing maC46 and GFP. On day 5 after transduction, cells were diluted with non-transduced cells to achieve 10% GFP^+^ cells, and the mixtures were infected with HIV-1_D117/II_. Data are presented as the mean ± the standard error of the mean (SEM) of triplicate samples. A. Flow cytometric analysis of the percentage of CD4^+^ T cells as a percentage of all viable cells. Infected cultures (closed symbols) showed a sustained loss of CD4^+^ T cells. B. Preferential survival of maC46-expressing CD4^+^ T cells following HIV-1 infection. C. Increased expression of GFP on PM-1 cells transduced with the maC46-GFP vector (M589) after HIV-1-infection. The geometric mean of GFP expression (Geo mean) on human CD4^+^ T cells was analyzed at the indicated time points.

We then analyzed if maC46 could also confer a selective advantage to primary human T lymphocytes in HIV-1-infected cultures. Primary human mononuclear cells were isolated, depleted of CD8^+^ T cells, prestimulated with anti-CD3 and anti-CD28-coated beads, and then transduced with the maC46-GFP vector M589 or the GFP control vector to a level of 80% and 57% GFP^+^ cells, respectively. On day 5 after transduction, cells were diluted with non-transduced CD8-depleted T cells to generate a transduction level of 10%. The cultures were challenged with the dual-tropic primary HIV-1 isolate D117/II. Cells were analyzed repeatedly by flow cytometry for CD4 and transgene (GFP) expression (Figure 5). In uninfected control cultures, the percentage of CD4^+^ T cells remained high throughout the 31 days of observation, and only slightly declined to 86% in the maC46 (M589) and 77% in the GFP control vector-transduced cultures. In contrast, the percent of CD4^+^ T cells declined drastically in the HIV-infected cell cultures. Cultures transduced with the control vector were completely deprived of CD4^+^ T cells by day 31 (Figure 5A). In the maC46-expressing, HIV-1-infected culture, loss of CD4^+^ T cells was much less pronounced, with the level of CD4^+^ T cells stabilizing at around 50% at day 20 (Figure 5A). The level of transgene-positive cells slowly declined in the non-infected and in the infected, control-vector transduced cultures. In contrast, the percentage of maC46-expressing cells increased to nearly 100% within 20 days in the HIV-1-infected cultures (Figure 5B). This accumulation of transduced cells was accompanied by an increase in the transgene expression level as measured by geometric mean fluorescence of GFP, which again declined after the percentage of transgene-positive cells increased after day 20, suggesting that the selective pressure of HIV-1 infection favored the survival of cells with high level transgene expression (Figure 5C).

### Positive selection of maC46-expressing primary human CD4^+^ T cells in a xenotransplant mouse model after HIV-1 infection

**Figure 6:**
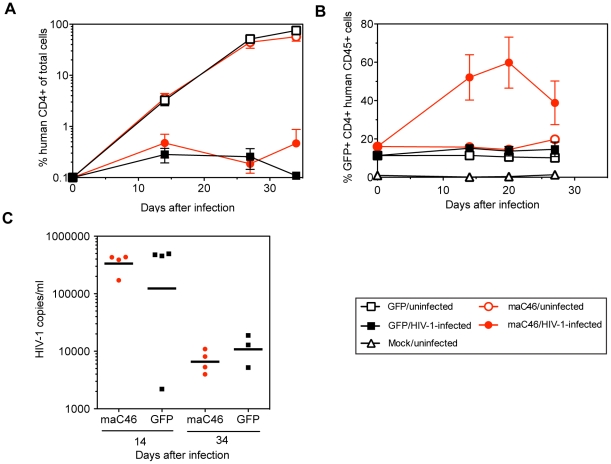
Survival advantage of maC46-expressing human CD4^+^ T cells in HIV-1-infected humanized mice. Human T cells were transduced with a control vector (M420) expressing GFP alone (squares) or the M589 vector expressing maC46-GFP (circles) and then infected with the HIV strain D117/II. Infected and uninfected cells were injected intraperitoneally into adult NOG mice. Four infected and 5 uninfected mice were followed for 5 weeks for each vector. A. Relative numbers of human CD4^+^ T cells. The percentage of human CD4^+^ T cells as a fraction of total viable cells was calculated. Means and the SEM for each group are shown. B. HIV infection leads to a selective survival of maC46-GFP expressing human CD4^+^ T cells (red closed circles) in vivo. Means and the SEM for each group are shown. C. Viral load in the serum of individual infected mice is not altered by expression of maC46-GFP. Differences in viral loads between mice receiving CD4^+^ T cells transduced with the maC46-expressing vector and the GFP control vector were not significant (Mann-Whitney test).

Finally, we studied the ability of maC46 to provide a selective advantage *in vivo* in a human xenotransplant mouse model. Human primary CD4^+^ T cells were transduced with maC46 (M589) or the lentiviral GFP control vector, infected with HIV-1_D177/II_, and transplanted into NOG (NOD SCID gamma_c_^−/−^) mice. Human CD4, human CD45, and GFP expression were monitored by flow cytometry. HIV-1 replication was analyzed by a commercial RT-PCR-based assay in mouse serum. When CD4^+^ T cell counts were examined as a percentage of all cells, CD4^+^ T cell counts in the blood of uninfected animals increased over time (Figure 6A), whereas in the HIV-1-infected animals the percentage of CD4^+^ T cells failed to expand in mice transplanted with CD4^+^ T cells expressing maC46 or in mice transplanted with cells transduced with the control vector. However, when the percentage of CD4^+^ cells was expressed as a percentage of human CD45^+^ cells, we observed a marked expansion of maC46-expressing cells from 18% up to 60% in the infected group during the first 20 days after transplantation (Figure 6B), while a stable level of transgene positive CD4^+^ T cells was seen in the uninfected mice and in the infected mice transplanted with cells expressing the control vector. Thus, maC46 is also able to confer a selective advantage in this *in vivo* setting. However, the percentage of maC46-expressing cells was not sustained and the level of transgene-positive cells declined again towards the end of follow-up. High levels of HIV-1 RNA of over 10^5^ copies per ml were measured in mouse sera at day 14, but viral loads decreased by 30 to 100-fold at day 34 (Figure 6C). No significant difference in viral load was seen between the maC46- and control vector treated groups, perhaps because sustained regeneration of maC46-expressing cells was not achieved in this xenograft model. At day 35, all mice developed severe xenogenic graft versus host disease and had to be euthanized.

## Discussion

The activity of three antiviral genes, an shRNA directed at *tat/rev*, a long RNA antisense element, and a membrane-anchored antiviral C peptide (maC46) were compared. All three transgene products suppressed HIV-1 replication in cell culture. However, only the membrane fusion inhibitor maC46 provided a strong selective advantage to gene-modified cells in infected T cell lines. Furthermore, maC46 led to the preferential survival of gene-modified cells in primary CD4^+^ T cells and in a xenotransplant mouse model.

All three antiviral genes have been previously shown to have potent antiviral activity, although the *tat/rev*-specific shRNA has been shown to mediate more robust inhibition of HIV when used in combination with other inhibitors [7], [9]–[11], [18], [25]. In addition, all of these inhibitors have all have been tested in clinical trials, either alone (VRX494, maC46) or in combination with other inhibitory genes (shI) [7], [9], [14], [18], [25]. Initially, we compared the ability of the three transgenes to suppress HIV-1 replication at different MOIs in cell lines, in which >95% of the cells were transduced. At a low MOI, a more pronounced antiviral effect was generally observed, especially early after infection. The most likely reason for this phenomenon is that the virus must go through more cycles of replication to infect all cells and reach saturation, the factor of inhibition (IF) being a function of the number of cycles (n) and the antiviral activity per cycle (i) (ideally IF = i^n^). In accordance with this prediction, the overall antiviral activity for the shRNA inhibitor and the antisense RNA was most pronounced at the lowest MOI. For the maC46 inhibitor, viral replication was found to be nearly completely suppressed, even at a high MOI. This is in accordance with the reported strong antiviral potency of membrane-anchored C-peptides of more than 1000-fold per viral entry cycle [26]. A variety of other approaches to inhibit HIV-1 entry have been developed, many of which target the CCR5 coreceptor [8], [27], [28], and future direct comparative studies with maC46 will be necessary to assess their relative potency. Taken together, while all genes showed antiviral activity, maC46 was most potent in this assay.

As noted above, the ability of an antiviral transgene to support selection of gene-modified cells may have a significant impact on its therapeutic activity, since only a small fraction of the estimated more than 10^11^ potential target cells for HIV-1 in a human subject can be genetically modified. In the setting of preferentially loss of unmodified CD4^+^ T cells due to HIV-induced cell death, expansion of CD4^+^ T cells expressing an inhibitor able to mediate a survival advantage could result in a repopulation of the immune system by cells resistant to HIV-1 infection, as was observed in the HIV-1-infected patient who underwent a bone marrow transplant with hematopoietic cells deficient in CCR5 [21]. However, in practice a number of factors may limit the ability of genetically-modified cells to expand and restore immune function *in vivo*, including the ongoing production of unmodified CD4^+^ T cells susceptible to HIV-1 infection, limitations in the proliferative capacity of modified cells, indirect mechanisms of cell death, and processes such as lymph node fibrosis that may affect the efficiency of CD4^+^ T cell reconstitution [29]. Although other mechanisms mediated by genetic inhibitors may also mediate therapeutic effects [12], [30], we found that the antiviral potency of the analyzed transgenes correlated with their ability to provide such a selective advantage. In mixed HIV-1-infected cultures of gene-modified and non-modified cells, only the maC46 expressing cells were able to expand. Transgene expression and antiviral activity is often more pronounced in cell lines than in primary T cells. Indeed, the level of maC46 on transduced primary T cells is consistently lower than on transduced T cell lines [9]. However, here we show that the level of transgene expression in primary human T cells is sufficient to support selection and sustained maintenance of gene-modified cells in *in vitro* cultures. Moreover, maC46-expressing human CD4^+^ T cells showed a preferential survival compared with untransduced CD4^+^ T cells after transplantation in HIV-1-infected NOG mice. However, the accumulation was only transient, the fraction of maC46-expressing cells declined towards the end of the observation period, and no effect on viral load was achieved. The observation that the maC46-expressing CD4^+^ T cells preferentially survived but did not proliferate and expand at the rate seen in uninfected mice suggests that T cell regeneration may be disturbed also for the uninfected, maC46-expressing CD4^+^ T cells in the infected mice. Unfortunately, the study of potential long-term effects of gene-therapeutic strategies on cell and virus dynamics is limited in this mouse model by the manifestation of xenogeneic graft versus host disease around week five after transplantation.

Despite the strong selective advantage conferred by maC46 found in this study, no significant accumulation of gene-modified peripheral CD4^+^ T cells was observed in a previous clinical trial in 10 HIV-1-infected patients that had failed HAART treatment [18]. Possibly, similar mechanisms counteracted the expansion of gene-protected cells in the presence of ongoing virus replication in the mouse experiment described here. These data show that a major challenge in gene therapy for HIV infection is to promote regeneration and expansion of the gene-protected CD4^+^ T cells under the selective pressure of ongoing virus replication, a hostile environment for T cell regeneration. Taken together, this study demonstrates the strong antiviral efficacy of maC46 as well as its ability to mediate a survival advantage *in vitro* and *in vivo*. Analysis of the ability of genetic inhibitors to mediate a survival advantage provides distinctive information not provided by standard inhibition assays and may offer information relevant to the *in vivo* efficacy of these genetic inhibitors.

## Materials and Methods

### Ethics statement

Animal experiments were performed in compliance with the local animal experimentation guidelines and approved by the regional council (Regierungspräsidium, Darmstadt, Germany, Protocol #F123/35). Human PBMCs were obtained from normal anonymous donors, who provided written informed consent under protocols approved by the Ethics Committee of the Medical Faculty of the Johann-Wolfgang Goethe University Frankfurt, Protocol #81/10.

### Cell culture

The cell lines CEMx174 (ATCC) and T2-SEAP cells (kindly provided by Welkin Johnson, NEPRC, HMS) were cultured in RPMI-1640 (Invitrogen, Carlsbad, CA) plus 20% fetal bovine serum (FBS, HyClone, Logan, UT), 10 mM HEPES, 50 U/ml penicillin and 50 µg/ml streptomycin (Cellgro, Mediatech, Manassas, VA), 2 mM L-glutamine (R20 medium) at 37°C with 5% CO_2_. The human embryonic kidney cell line 293T and the human osteosarcoma cell line U20S were cultured in DMEM (Invitrogen) plus 10% FBS, 50 U/ml penicillin and 50 µg/ml streptomycin, 2 mM L-glutamine (D10 medium) at 37°C with 5% CO_2_. The cell line PM-1 (obtained through the NIH AIDS Research and Reference Reagent Program, Division of AIDS, NIAID, NIH from Drs. Paulo Lusso and Robert Gallo) was cultured in RPMI 1640 supplemented with 5% fetal bovine serum, 2% L-glutamine and 1% penicillin/streptomycin and the cell lines A3.01-CCR5 (EU Programme EVA Centre for AIDS Reagents) in RPMI 1640 with 5% fetal bovine serum, 2% L-glutamine, 1% penicillin/streptomycin and 1 mg/ml G418 (Roth, Karlsruhe, Germany).

Human low-density mononuclear cells were obtained from normal anonymous donors, and were isolated by density gradient centrifugation (Pancoll, PAN, Aidenbach, Germany), treated with 0.45% ammonium chloride to lyse red blood cells and washed with PBS. CD8^+^ cells were depleted using CD8-specific microbeads (Miltenyi Biotec, Bergisch Gladbach, Germany). PBMCs were stimulated with Dynabead CD3/CD28 T cell expanders (Dynabeads CD3/CD28, Invitrogen Dynal, Eggenstein, Germany) and cultured in X-Vivo 15 with 5% human serum, 2% L-glutamine, 1% penicillin/streptomycin, 20 mM HEPES and recombinant human IL-2 (100 U/ml, Proleukin, Novartis, Berne Switzerland) at 37°C with 5% CO_2_.

### Lentiviral vectors

The construct maC46 (M589) was generated using a three step PCR. For the first PCR reaction, the maC46 transgene cassette was amplified from M87o-RRE [9] using the oligonucleotides M87o-for (5′ GGG GGA TCC CCC GGG CTG CAG GAA TTC GCC CTT CTC TAG CGC TAC CGG TCG CCG C 3′) and M87o-rev (5′ TCC TCG CCC TTG CTC ACC ATG CAT GCG GGC TCC AGC TCC AGG CGC T 3′). In a second PCR reaction, the GFP sequence was amplified from pHR9SIN.cPPT-SEW [31] (M420) using the oligonucleotides GFP-for (5′ GCC CGC ATG CAT GGT GAG CAA GGG CGA GGA G 3′) and GFP-rev (5′ CTA CTA GAT ATC GAA TTC ACA TGT GGT GGT GGT GGT GG 3′). Finally, the two PCR products were used in a third PCR to create a cassette containing maC46 fused to GFP. This PCR product was digested with EcoRV and BamHI. pHR9SIN.cPPT-SEW was digested with SbfI, the 3′ overhang was removed, and the vector was further digested with BamHI to remove the GFP transgene and finally ligated with the maC46-GFP cassette.

Virions for the lentiviral vector VRX494 [32] were generated at VIRxSYS (Gaithersburg, MD) by transient transfection of 293T cells with the transfer vector and a plasmid that expresses Gag-Pol, Tat, Rev and VSV-G as previously described [25]. Dilutions of viral stock were used to transduce HeLa-Tat cells and the percentage of GFP positive cells was determined by flow cytometry. Stock titers ranged from 2.8×10^9^ to 4×10^9^ transducing units (TU) per ml.

Virions for the lentiviral vectors HJ57, pHIV-shI-GFP [10], [11], M420 (GFP lentiviral control used for PBMC and mouse experiments) and maC46(M589) were generated by transient transfection of 293T cells in a five plasmid system with the transfer vector and separate plasmids that expresses Gag-Pol (pHDM-Hgpm2), Tat (pHDM-tat1b), Rev (pRC-CMV rev1B) and VSV-G (pHDM VSV-G) or in a three plasmid system with the transfer vector and separate plasmids that express Gag-Pol, Tat and Rev (pCMV-dR8.91 [33]) and VSV-G (pHCMV-VSV-G [34]) in D10 medium supplemented with sodium benzoate. Replication-deficient HIV-1 vector particles expressing GFP were produced by transient transfection of 293T cells using plasmids that express GFP (M420), Gag Pol, Tat and Rev (pCMV-dR8.91) and JRFL HIV-1 envelope [35]. Supernatants were clarified by filtration, and virions were concentrated by ultrafiltration. Virus stocks were analyzed for HIV p24 Gag by ELISA (Coulter HIV-1 Core Antigen Assay, Coulter International Corp., Miami, FL; or Advanced BioScience Laboratories, Inc., Kensington, MD) according to the manufacturer's instructions. Dilutions of viral stock were used to transduce CEMx174 cells or U20S cells or PM-1 cells and the percentage of GFP positive cells was determined by flow cytometry. Stock titers were generally around 10^7^ per ml.

### Transductions

CEMx174 cells were transduced with the lentiviral vectors HJ57, pHIV-shI-GFP, and M589 at MOIs of 0.5 IU per cell in R20 medium plus 8 µg/ml polybrene (Sigma) during 30 min spinoculation (2000 rpm) and subsequent overnight culture at 37°C with 5% CO_2_. After 24 hours, the cells were washed and expanded in R20 media. The CEMx174-VRX494 cells were transduced as previously described [36]. Human PBMC were isolated via density gradient centrifugation (Pancoll, PAN, Aidenbach, Germany) and CD8-depleted using human CD8 microbeads (Miltenyi Biotec). Primary human T lymphocytes (5×10^6^ cells per 6-well in 5 ml medium) were transduced with M589 or M420 at MOI of 3 and stimulated with beads (Dynabeads CD3/CD28, Invitrogen Dynal, Eggenstein, Germany). After 3 days cells were washed with medium, reseeded in fresh medium (1×10^6^ cells/ml) and further cultivated for 2 days. On day 5 magnetic, beads were removed, cells were diluted with non-transduced cells to attain a final concentration of 10% GFP^+^ cells and used for experiments.

Vector-transduced CD4^+^ T cells were analyzed for the presence of replication-competent lentivirus by incubating supernatant from transduced cells with T2-SEAP cells, which have Tat-dependent expression of secreted embryonic alkaline phosphate (SEAP). Three-five days later, SEAP expression in cell-free supernatant was measured using the Phospha-light system (Tropix, Applied Biosystems, Foster City, CA). Based on experiments with HIV-1_NL4-3_, this assay has a lower limit of detection of 50 infectious particles per ml. All assays for replication-competent lentivirus were negative.

### Flow cytometric analysis

Transduced populations of CEMx174 cells were analyzed for the percentage of GFP expression in each population by flow cytometry using a FACSCalibur (BD BioSciences, San Jose, CA). GFP expression was measured in the FITC channel. Transduced populations of CEMx174 cells (GFP^+^ cells) were sorted to greater than 95% GFP^+^ using a BD FACS Vantage. PBMCs were resuspended in PBS with 1% FCS and 0.05% sodium azide (FACS buffer) and stained by incubation with human-specific PerCP-conjugated anti-human CD4 antibodies (clone SK3, BD-PharMingen, Heidelberg, Germany) for 15 minutes at room temperature. Cells were analyzed by flow cytometry using a FACSCalibur.

Expression of GFP and intracellular expression of HIV-1 p24 Gag was determined in transduced and infected CEMx174 and PM-1 populations using Perm & Fix reagents (Caltag Laboratories, Burlingame, CA) and staining with a PE-conjugated HIV p24 Gag antibody [37] (Clone KC57, Beckman Coulter, Brea, CA). The cells were washed and analyzed for expression of GFP and HIV-1 Gag by flow cytometry using a FACSCalibur.

To analyze transgene expression in the different cell populations *in vivo* approximately 50 µl blood or splenocytes of transplanted mice were incubated with 1 µg of a monoclonal antibody to mouse Fc-receptors (2.4G2, Bio Express, West Lebanon, NH) for 15 minutes at room temperature. Cells were stained with PerCP-conjugated anti-human CD4 antibodies (clone SK3, BD-PharMingen) and APC-conjugated anti-human CD45 antibodies (clone HI30, BD-PharMingen) for 15 minutes at room temperature. Erythrocytes were lysed using lysing buffer (BD PharmLyse). Cells were washed and analyzed using a FACSCalibur.

### Viral replication assays

HIV-1_NL4-3_ viral stocks (kindly provided by Ronald C. Desrosiers, NEPRC, HMS) were generated by infection of CEMx174 cells and harvesting cell free supernatants on day 8–12 after infection. Viral production was analyzed by ELISA for HIV-1 p24 Gag (as described above) and/or titered for TCID_50_ by limiting dilution assay targeting T2-SEAP cells with slight modifications [38]. HIV-1_D117/II_ is a highly cytopathic dual-tropic primary isolate generated at the Georg-Speyer-Haus (von Briesen, unpublished data), which was used for the experiments in primary T cells, as it readily replicates in PBMC. HIV_D117/II_ viral stocks were generated by infection of human PBMC. Cells were passaged for 1–2 weeks and cell free supernatants were harvested. Viral stocks were titrated on TZM-bl cells as described previously [39]. Vector-transduced and untransduced CEMx174 cells and mixed populations of vector-transduced and non-transduced CEMx174 cells were resuspended in viral supernatant at MOIs of 0.0001 to 0.01 TCID_50_/cell of HIV-1_NL4-3_ for 4 hours, before washing and initiating cultures in 10–20 ml R20 media. Viral replication was assessed by measuring p24 Gag production in cell-free supernatant with ELISA as described above. The percentage of GFP^+^ and/or HIV-1 p24 Gag^+^ cells was analyzed by flow cytometry.

For primary human T lymphocytes cells were transduced as described above, and on day 5 after transduction, cells were infected with HIV_D117/II_. After 16 hours cells were washed with PBS and reseeded in fresh medium in 24 well plates (5×10^5^ cells per well). Every 2–3 days, cells were split and fresh medium with 100 U IL-2/ml was added.

### Localization of p24 via confocal microscopy

Mixed populations of maC46-GFP and native PM-1 cells were infected with HIV-1D117/II. Cells were stained intracellularly for p24 antigen (see above). Cells were spun on cover slips using a cytospin (Tharmac CellSpin II, Tharmac, Waldsolms, Germany). The cover slips were fixed on the microscope slide using mounting medium (ProLong Gold antifade reagent, Molecular Probes-Invitrogen, Karlsruhe, Germany) and then analyzed with a confocal microscope (Leica TCS SL with Leica LCS-Software, Leica Microsystems, Wetzlar, Germany) for p24 and GFP localization.

### Virus replication assays in xenotransplanted NOG mice

NOD/SCID/gamma chain knockout mice (NOD.Cg-PrkdcscidIl2rgtm1WjL/SzJ) were obtained from Charles River laboratories (Sulzfeld, Germany). Mice were bred and maintained under specific pathogen free conditions in individually ventilated cages in the animal facilities of the Georg-Speyer-Haus. The experiments were performed in compliance with the local animal experimentation guidelines. On day 5 after transduction, human T lymphocytes were infected with HIV_D117/II_. After 16 hours cells were harvested, washed with PBS and transplanted into adult NOG mice via intraperitoneal injection. Blood of the transplanted mice was regularly collected from the tail vein. Viral load in the serum of the infected mice was determined by RT-PCR (AmpliPre Cobas Taqman, Roche, Basel, Switzerland). Mice were euthanized after anesthesia by cervical dislocation as soon as they showed signs of severe graft versus host disease.

### Statistical analysis

Statistical analysis were done using GraphPad Prism software (GraphPad Software, Inc., La Jolla, CA).
